# Ultra-High T_g_ Thermoset Fibers Obtained by Electrospinning of Functional Polynorbornenes

**DOI:** 10.3390/nano12060967

**Published:** 2022-03-15

**Authors:** Basile Commarieu, Moubarak Compaoré, Raphaël de Boëver, Régis Imbeault, Maxime Leprince, Barbara Martin, Bruno Perard, Weiguang Qiu, Jerome P. Claverie

**Affiliations:** Quebec Center for Functional Materials, Department of Chemistry, Université de Sherbrooke, 2500 Blvd de l’Université, Sherbrooke, QC J1K2R1, Canada; basile.commarieu@saint-gobain.com (B.C.); moubarak.ca@outlook.com (M.C.); raphael.de.boever@usherbrooke.ca (R.d.B.); regis.imbeault@usherbrooke.ca (R.I.); maxime.leprince@usherbrooke.ca (M.L.); barbara.martin@usherbrooke.ca (B.M.); bruno.perard@usherbrooke.ca (B.P.); weiguang.qiu@usherbrooke.ca (W.Q.)

**Keywords:** electrospinning, thermoset, nanofiber, epoxy, nanoparticles

## Abstract

Insertion polynorbornenes (PBNEs) are rigid-rod polymers that have very high glass transition temperatures (T_g_). In this study, two functional PNBEs were electrospun in the presence of a variety of cross-linkers, resulting in fibers with T_g_s greater than 300 °C. The fibers are long (several mm), rigid, and with diameters that can be tuned in the range 300 nm–10 μm. The electrospinning process can be used to encapsulate dyes or graphene dots. Due to the high cross-linking density of the fiber, dye leaching is prevented. In contrast with other rigid-rod polymers, electrospinning of PNBE is facile and can be performed at injection rates as high as 1 mL/min.

## 1. Introduction

Electrospinning is a versatile method to prepare fibers with diameters typically ranging from 100 nm to 10 µm [1]. Their size, morphology, and alignment can be conveniently tuned by adjusting operational parameters such as voltage, injection speed, solvent or injector, and collector configuration [2]. Owing to its amazing flexibility and simplicity, electrospinning has attracted much attention, both in academic and industrial environments [3,4,5]. Many promising applications stem from the incorporation of nanoparticles into the fibers to prepare functionalized nanocomposite fibers with potential applications in catalysis, sensing, drug delivery, and energy-related materials [6,7]. Virtually any polymer can be electrospun, with a vast body of research turned toward polymers with glass transition temperature, T_g_, or melting temperature, T_m_, lower than 200 °C. The resulting fibers cannot be heated past this temperature without losing their high aspect ratio, thus greatly limiting the use of electrospun fibers for high-temperature applications. In order to improve the mechanical properties of the fibers, cross-linking can be performed during electrospinning [8]. Alternatively, physical cross-linkers that improve the electrospinning of unentangled polymers can be added to the formulation [9]. However, electrospinning of high-performance polymers such as polyimides [10,11], polyaramides [12,13], or benzoxazole [14,15,16] presents operational difficulties, which have offset the unique advantages of the resulting fibers, such as high thermal resistance, high T_g_, and excellent mechanical properties. For example, poly(p-phenylene terephtalamide) (known as Kevlar©) is electrospun in concentrated sulfuric acid [12]. Many aromatic prepolymers resist dissolution due to the presence of extensive H-bond networks unless chaotropic salts such as LiCl are added [13,14,15,16]. These salts eventually need to be removed from the fibers, for example, by using Soxlet extraction [17], as their presence reduces H bonding within the fiber, resulting in a deterioration of the mechanical properties and an increase in the swelling by water. Lastly, fibers of polyimides [11] and polybenzoxazoles [18] must be cured at temperatures above 300 °C in order for imidization to occur, a step that is necessary for the polymer to reach high T_g_. 

**Figure 1:**
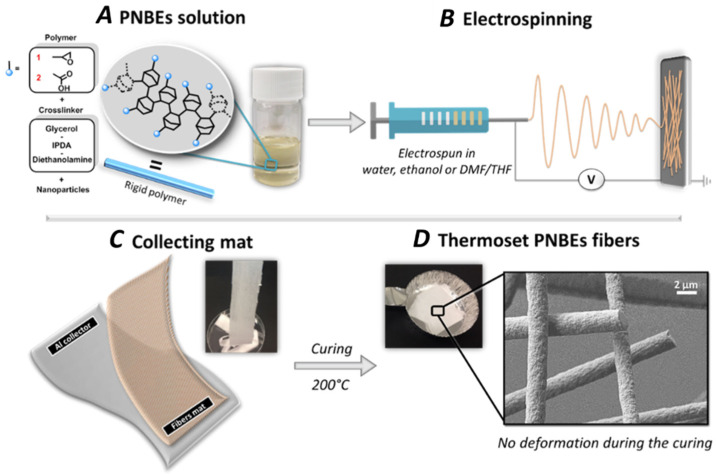
(**A**) Functional PNBE electrospun in this study; (**B**) schematic electrospinning process; (**C**) fibers collection process; (**D**) SEM image of the functional PNBE fibers after curing.

Recently, we have shown that insertion polynorbornenes (PNBE) [19,20] prepared by catalytic polymerization of functionalized norbornenes [21,22] can lead to ultra-high T_g_ thermosetting materials (Figure 1). Two main polymers were presented. The first one, a PNBE bearing an epoxy pendant group (polymer **1**, Figure 1), could be cross-linked using a variety of cross-linkers such as glycerol, maleic anhydride, or isophorone diamine (IPDA). The resulting thermosets exhibited T_g_s above 340 °C, temperatures at which the thermoset started to decompose. Alternatively, a PNBE bearing pendant carboxylic acid groups (polymer **2**, Figure 1) could easily be cross-linked with butanediol diglycidyl ether (BDE), leading to thermosets with T_g_s above 350 °C. In this report, we demonstrate that both polymers **1** and **2** can be very simply electrospun, in a variety of solvents, including environmentally friendly ethanol and water. The resulting fibers are easily cross-linked, leading to individual thermoset nanofibers with unique thermal resistance and ultra-high T_g_. We also demonstrate that the electrospinning process is convenient for the fabrication of nanocomposite hybrid fibers containing graphitic dots, GDs, leading to fibers that are fluorescent. Interestingly, electrospun fibers containing GDs have been shown to exhibit antibacterial properties [23]. Thus, these preliminary results indicate that PNBE polymers are promising candidates for the generation of high-T_g_ nanofibers with properties that compare with those of conventional high-performance polymers. Nano- and microfibers with high-temperature resistance have important applications for protective clothing against heat [24]. In particular, heat resistance is a desirable property for protective clothing, when working in environments with high heat radiation. For such applications, polyaramid, polytetrafluorethylene, and polyimides are usual candidates [24,25]. We envision that PNBE polymers that are stable up to 300 °C, that are readily electrospun at rates as high as 1 mL/min and that can be easily formulated offer an interesting alternative to these other polymers that are difficult to electrospin and formulate. 

## 2. Materials and Methods

Polymers **1** and **2** were prepared using the method reported in [19,20]. Cross-linkers such as IPDA, BDE, and diethanolamine (DEA) were used. Solvents such as dimethylformamide (DMF) and tetrahydrofuran (THF) were purchased from Sigma Aldrich (Burlington, MA, USA) and used without any further purification. The GDs were prepared using the method reported in [26,27]. 

### 2.1. Characterizations 

The molecular weight distributions of the polymers were determined by gel permeation chromatography (GPC) using an Agilent instrument (Santa Clara, CA, USA) with triple detection and equipped with three PL Gel 10 μm Mixed B columns. For polymer **1**, elution was performed in THF while it was performed in basic water (100 mM NaNO_3_, 50 mM LiCl, 50 mM NaHCO_3_, 20 mM Et_3_N, 5 mM NaN_3_). Differential scanning calorimetry (DSC) measurements of solid polymers were performed on a DSC823e (TOPEM modulation, Mettler Toledo, Greifensee, Switzerland) equipped with an FRS5 sample cell, a sample robot, a Julabo FT400 intracooler (Seelbach, Germany), and an HRS7 sensor from Mettler Toledo (Greifensee, Switzerland). Samples were heated from 50 °C to 400 °C at a rate of 10 °C/min, and data were analyzed with STAR software. The data associated with the second heated ramp are shown. The solid polymers Fourier transformed infrared, FTIR, spectra of the fibers were recorded on a Nicolet 6700 Spectrometer equipped with Smart attenuated total reflectance accessory (ThermoSci, Waltham, MA, USA). The samples were analyzed by thermogravimetric analysis (TGA), using a TA Q500 instrument (New Castle, DE, USA) coupled to a Discovery MS, with a temperature range from 30 to 1000 °C at a 20 °C/min rate. Fibers were analyzed by scanning electron microscopy (SEM) on a Hitachi S-4700 SEM (Chiyoda, Tokyo, Japan) instrument equipped with an energy-dispersive X-ray analyzer (EDX). Fibers containing CNTs were analyzed by transmission electron microscopy (TEM) using a Jeol JEM-2100F (Akishima, Tokyo, Japan) microscope equipped with a field-emission gun running at 200 keV. Fiber diameter was measured at 20 different locations (excepted for fiber 1, for which *ca* 150 measurements were performed), and the average is reported in Table 1. To prepare the sample, the fibers were ground with a spatula, and water was added to partially disperse the fibers. A drop of this suspension was deposited on a Lacey grid (mesh200). The grid was dried overnight before analysis. 

### 2.2. Co-Electrospinning of Polymer 1 with IPDA Cross-Linker (Typical Experiment)

In a flask, 2 g of polymer **1** was dissolved in 5 g of DMF at 60 °C on the orbital shaker. Immediately before electrospinning, at room temperature, 0.66 g of IPDA were mixed into this polymer solution. The resulting solution was introduced in a glass syringe equipped with a 0.41 mm diameter flat needle. A 20 kV positive voltage was applied to the needle tip using a CZE 1000R high-voltage power supply (Spellman High Voltage Electronics, Hauppauge, NY, USA), while a 2 kV negative potential (Power Designs, Danbury, CT, USA) was imposed on two parallel metallic rods to collect the electrospun fibers. The distance between the needle tip and the collector was 10 cm. The speed of injection was 0.1 mL/min. Curing of the fibers was performed by leaving the fibers 2 h at 200 °C. Table 1 consigns the various conditions used for the preparation of each fiber. 

### 2.3. Electrospinning of Polymer 2 (Typical Experiment)

In a flask, 2 g of polymer **2** was dissolved in 3.5 g of ethanol at 60 °C on the orbital shaker. The resulting solution was introduced in a glass syringe equipped with a 0.41 mm diameter flat needle. A 20 kV positive voltage was applied to the needle tip using a CZE 1000R high-voltage power supply (Spellman High Voltage Electronics), while a 2 kV negative potential (Power Designs) was imposed on two parallel metallic rods to collect the electrospun fibers. The distance between the needle tip and the collector was 20 cm. The speed of injection was 0.5 mL/min. Curing of the fibers was performed by leaving the fibers 2 h at 200 °C.

## 3. Results and Discussion

In this study, functional insertion PNBEs obtained by catalytic vinyl polymerization were used to generate the thermosets fibers. Recently, we have reported a straightforward synthesis of functional PNBEs **1** and **2 [19,20,21,22]**, whereby readily available raw materials such as dicyclopentadiene, butadiene, and acrylic acid were converted into polymers **1** and **2** in three and two steps, respectively. It should be noted that these functional PNBEs have different structures from more common PNBEs obtained by ring-opening metathesis polymerization (ROMP). Functional insertion PNBEs are saturated, chemically stable, and have extremely high T_g_s (typically above 350 °C), whereas ROMP PNBEs are unsaturated, are prone to air oxidation, and have low T_g_s (below or around room temperature). 

Unlike other polyolefins, functional insertion PNBEs are readily soluble in variety of solvents (Table S1) and they have a moderate molecular weight (*M_n_* = 19,600 g/mol and *M_w_* = 41,100 g/mol for **1**, *M_n_* = 18,500 g/mol and *M_w_* = 29,400 g/mol for **2**, as measured by GPC with absolute molecular weight determination). These polymers adopt a rigid-rod conformation in solution as shown by the Mark–Houwink exponent, as determined by viscosimetry (using the online viscosimeter of the GPC). Indeed, the intrinsic viscosity measured at 40 °C are given by

$$\left[\eta \right]=1.82\times 10^{-5}M_{w}{}^{0.89}\;\mathrm{in}\;\mathrm{dL}/g\;\mathrm{in}\;\mathrm{dL}/g\;\mathrm{for}\;\mathrm{polymer}\;1\;\mathrm{in}\;\mathrm{THF}$$

$$\left[\eta \right]=2.55\times 10^{-5}M_{w}{}^{0.85}\;\mathrm{in}\;\mathrm{dL}/g\;\mathrm{in}\;\mathrm{dL}/g\;\mathrm{for}\;\mathrm{polymer}\;2\;\mathrm{in}\;\mathrm{basic}\;\mathrm{water}$$

**Figure 2:**
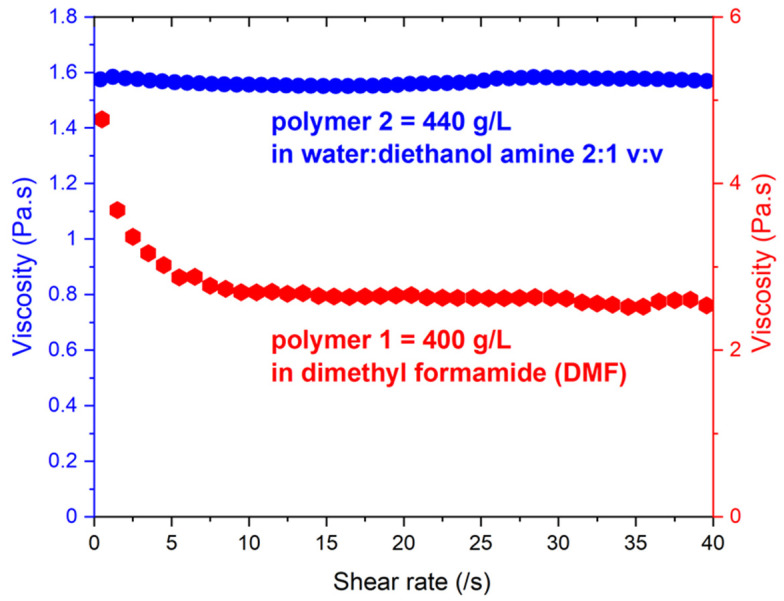
Viscosity of polymer solutions vs. shear rate (using polymer concentrations that correspond to electrospinning conditions, as shown in Table 1).

Since these polymers have a moderate molecular weight, as well as a rigid-rod-like conformation, they are not prone to entangle, and their viscosity in solution remains low (Figure 2) even at concentrations as high as 400 g/L. 

**Figure 3:**
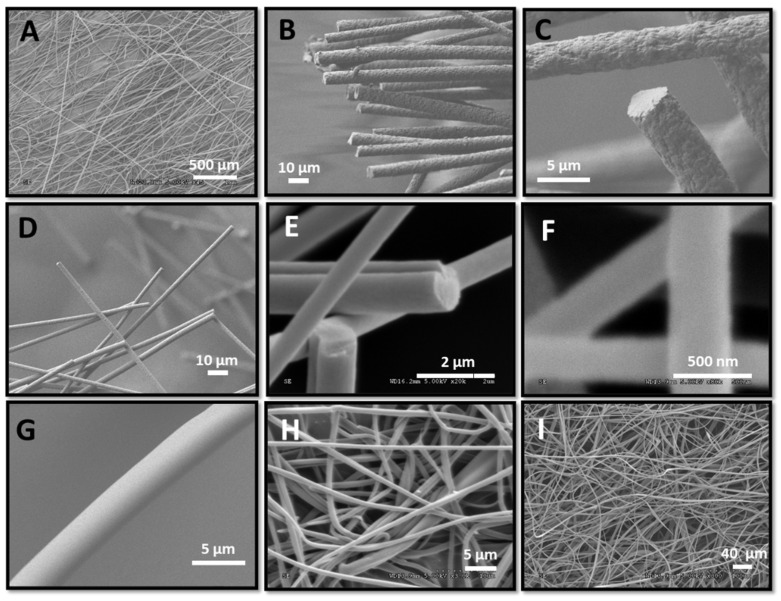
SEM pictures of the fibers (**A**) expt. 1. (**B**) expt 2. (**C**) expt. 3. (**D**) expt 4. (**E**) expt 5. (**F**) expt 6. (**G**) expt 7. (**H**) expt 8. (**I**) expt 12.

**Figure 4:**
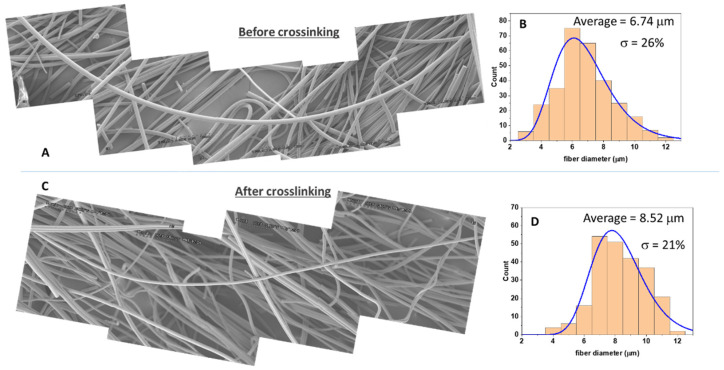
Assembled SEM pictures of fibers obtained by expt 1 (Table 1) before (**A**) and after (**C**) thermal treatment. The fiber that crosses all the images is approx 1 mm long. Each individual image has 490 × 220 μm dimension. Histogram of the fiber diameters measured on 150 fibers, measured before (**B**) and after (**D**) thermal treatment.

We have shown in the past that thermal treatment of polymer **1** or **2** and a suitable cross-linker at temperatures of 150–200 °C leads to the formation of highly cross-linked thermosets with T_g_s as high as 350 °C. In this study, the cross-linker selected for polymer **1** was IPDA, whereas glycerol, diethanolamine, and diglycidyl ether were used as cross-linkers for polymer **2**. The mixture of solvent, polymer, and cross-linker was electrospun using various experimental conditions and either a rotative or static collector, and cross-linking was performed by curing the resulting fibers (Figure 1). Importantly, the polymer and cross-linker solution were stable (no measurable increase in viscosity) at room temperature for at least several hours. Polymer **1** and cross-linker IPDA (expts 1–6, Table 1) were electrospun to form fibers of 0.4–8 µm diameter (Figure 3). Notwithstanding their composition, the fibers were soluble immediately after electrospinning, but they became completely insoluble after curing at ca. 200 °C for 2 h. Several solvents were assessed (DMF, THF, ethanol, and water), and in all cases, the fibers did not swell even once cross-linked (Table S1). Upon heating, cross-linking occurred, as shown by the disappearance of the epoxy band at 874 cm^−1^ and by the appearance of the alcohol band around 3500 cm^−1^ using FTIR spectroscopy (Figures S1–S6). The aspect ratio of the fibers was not affected by the cross-linking process, as shown by comparing fibers before and after cross-linking based on SEM results (Figure 4); however, a small diameter increase was observed after curing (Figure 4B vs. Figure 4D). These fibers were long, with most fibers measuring several millimeters in length (Figures S1–S6 and Figure 4). The fibers were rather monodisperse, with a relative standard deviation of around 20% (Figure 4B,D), measured over 200 fibers. 

Based on SEM results, these fibers appeared as rigid rods when observed at a 100 um length scale or below (Figure 3B–F). Such rigidity stems from the high rigidity of the PNBE fibers. Based on DSC results, no thermal transition such as T_g_ or T_m_ could be located in the temperature range 25–300 °C, indicating that these fibers remain in their glassy state, even at such high temperatures. The unique rigidity of these fibers was also revealed by the characteristic brittle fracture (Figure 3B–E). Furthermore, these fibers did not show any sign of thermal decomposition (Figure S7) or yellowing/charring before 300 °C (Figures S1–S6). Interestingly, the fiber diameter could easily be adjusted by changing the ratio of polymer to cross-linker (expts 1–4), with greater cross-linker concentrations leading to smaller fibers. Since such behavior was also observed for the fibers prior to cross-linking, we attribute this phenomenon to a solvent effect. In electrospinning, fiber diameters are highly dependent on the solvent nature [1], and the cross-linker acts as a cosolvent when the electrospinning experiment is initiated. Fiber diameters could also be adjusted by tuning the injection rate (expts 3, 5, and 6). Thus, by changing the injection rate from 0.03 mL/min to 0.1 mL/min, the diameter varied from 400 nm to 5 µm.

To demonstrate the versatility of the PNBE platform, polymer **2** was also electrospun in the presence of various cross-linkers, such as diethanolamine, glycerol, and DGE. This polymer is electrospinable in DMF (expt 8), as well as in more environmentally friendly solvents such as ethanol or water (expts 7, 9–12). Cross-linking was performed at temperatures ranging from 150 to 200 °C. Once again, the fiber diameter could be tuned from 300 nm to 3 µm, either by changing the composition or the injection speed (expts 9–12). Similar to polymer **1**, the fibers were soluble before cross-linking but totally insoluble after cross-linking (Table S1), and they exhibited remarkable thermal stability up to 300 °C, as shown by optical microscopy observations (Figures S8 and S9). Based on FTIR results (Figures S9 and S10), it can be inferred that cross-linking occurred due to the formation of ester and/or amide groups between the polymer and the cross-linker. 

**Figure 5:**
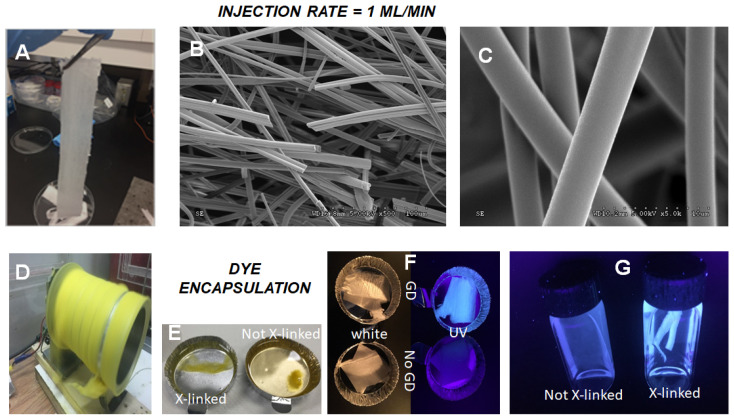
Mat of fibers (**1**:IPDA = 83:17 in DMF) obtained at high injection rate (1 mL/min) (**A**) and SEM pictures of the fibers (**B**,**C**). Cylindrical collector with fibers containing a yellow dye after 5 min of electrospinning (**1**:IPDA = 66:33 in DMF, injection = 1 mL/min) (**D**) and same fibers immersed in THF ((**E**) before and after cross-linking). Pictures of fibers containing GDs (**1**:IPDA:GD = 82:16:2 in DMF electrospun at 1 mL/min) under white and UV light (**F**) compared with fibers devoid of GDs. GD-containing fibers before and after cross-linking immersed in THF, under UV light (**G**).

The electrospinning of these rigid thermoset PNBE formulations proved to be remarkably easy. For example, it was possible to electrospin at high injection rates (1 mL/min), leading to the rapid formation of a mat (Figure 5A–C) constituted of fibers with diameters 11 μm (Video S1, Supporting Information). Such high injection rates are usually not possible in electrospinning or at least are known to lead to beading and irregular fibers. In our case, we believe that the low viscosity of the polymer solution, as well the low propensity to entanglement, allows for the rapid injection of the polymer solution within the electrical field. After curing, the fibers were completely cross-linked, and they were impervious to swelling by water or any organic solvent. Thus, we explored the feasibility of permanently encapsulating dyes within the fibers. Dye leaching is a major issue that affects most dyed textiles, leading to durability issues in the clothing industry, as well as pollution issues near textile factories. Dye immobilization, a process in which the dye is fixed to the fiber using covalent interactions, can be used as a potential solution to dye leaching issues [8,28]. However, such a strategy requires at least one additional synthetic step, while many dyes are devoid of easily conjugated moieties. We sought to explore whether dye encapsulation within a cross-linked fiber offered a potentially simpler alternative to dye immobilization by covalent bonding. For example, a yellow dye was co-electrospun with polymer **1**, using IPDA as a cross-linker (Figure 5D). In this case, the yellow fibers were found to immediately release the dye in THF before cross-linking (Figure 5E). By contrast, after cross-linking, no dye leaching was observed, even after several hours of immersion in THF. This experiment was successfully repeated with a purple dye (Video S1). The same electrospinning procedure was also used to prepare fibers containing fluorescent nanoparticles such as GDs, with an average diameter of 2.1 nm. Once again, electrospinning at a 1 mL/min injection rate led to rigid fibers with 8.9 μm diameter (Figure S11), which were initially soluble in THF but totally insoluble after cross-linking (Figure 5E). Interestingly, the presence of the blue GDs in the fibers was inconspicuous under white illumination but clearly apparent under UV light (Figure 5F). The fact that the GDs were not leached by the solvent (Figure 5G) in the cross-linked fiber indicates that the GDs were incorporated within the fiber, not merely adsorbed. Thus, these fluorescent fibers can eventually be used as durable security devices with fluorescent markings. 

## 4. Conclusions

In this study, we demonstrated that fibers with T_g_ above 300 °C can be easily prepared by electrospinning (it is worth noting that T_g_ could not precisely be determined because the fiber began to degrade above this temperature). Despite being brittle (as shown by their fractures in Figure 3), these fibers formed a continuous non-woven mat, which is similar to any textile mat. Remarkably, injection rates as high as 1 mL/min could be used to prepare the fibers. Such a surprising result can be explained by the low propensity of entanglement for solutions of rigid-rod polymers. Once cross-linked, the fibers did not swell and became insoluble. Thus, by co-electrospinning polymer **1** with various dyes, we were able to permanently encapsulate the dye and prevent leaching. For example, GDs were successfully encapsulated within the PNBE nanofibers, with no observed leaching once the fibers were cross-linked. Due to their unique photophysical properties, we believe that GD-containing PNBE fibers could potentially become useful photocatalysts and be incorporated into antibacterial textiles [23]. Thus, we believe that functional PNBEs are promising candidates for the fabrication of functional fibers with high T_g_.

## Figures and Tables

**Table 1:** Electrospinning conditions to form various PNBE-based thermoset fibers.

Expt	Polymer	Cross-linker ^1^	Mass Ratio ^2^	Solvent	Injection ^3^ (mL/min)	Diameter ^4^ (µm)
1	**1**	IPDA	83:17	DMF/THF	0.1	8.5
2	**1**	IPDA	75:25	DMF/THF	0.1	5.7
3	**1**	IPDA	67:33	DMF/THF	0.1	4.6
4	**1**	IPDA	50:50	DMF/THF	0.1	2.2
5	**1**	IPDA	67:33	DMF/THF	0.05	1.0
6	**1**	IPDA	67:33	DMF/THF	0.03	0.4
7	**2**	DEA	67:33	Water	0.5	1.6
8	**2**	DEA	85:15	DMF	0.002	0.3
9	**2**	Glycerol	67:33	Ethanol	0.5	2.9
10	**2**	DGE	60:40	Ethanol	0.01	0.5
11	**2**	DGE	60:40	Ethanol	0.03	0.6
12	**2**	DGE	60:40	Ethanol	0.05	0.8

^1^ IPDA: isophorone diamine, DEA: diethanolamine, DGE: butanediol diglycidyl ether. ^2^ Composition of polymer to cross-linker (wt:wt). ^3^ Injection rate. ^4^ Average fiber diameter, as measured by SEM for fibers that were cured.

## Data Availability

Not applicable.

## Supplementary Materials

The following supporting information can be downloaded at: https://www.mdpi.com/article/10.3390/nano12060967/s1, Figure S1: Pictures of fibers obtained in experiment 2; Figure S2: Fibers obtained in experiment 2 before and after heat treatment. (Optical microscopy and FTIR); Figure S3: Pictures of fibers obtained in experiment 3; Figure S4: Fibers obtained in experiment 3 before and after heat treatment. (Optical microscopy and FTIR); Figure S5: Pictures of fibers obtained in experiment 4; Figure S6: Fibers obtained in experiment 4 before and after heat treatment. (Optical microscopy and FTIR); Figure S7: TGA analysis of the fibers prepared in experiment 5; Figure S8: Pictures of fibers obtained in experiment 7; Figure S9: Fibers obtained in experiment 7 before and after heat treatment. (Optical microscopy and FTIR); Figure S10: Zoom of the FTIR spectra of (from top to bottom): fibers from experiment 7, fibers from experiment 7 heated at 200 °C for 30 min, polymer **2**, diethanol amine; Figure S11: SEM pictures of fibers containing GDs. Table S1: solubility of the fiber, Video S1: electrospinning of polymer 1, IPDA, and a purple dye at 1 mL/min.
