# Supplementary material for: Ultra-High Tg Thermoset Fibers Obtained by Electrospinning of Functional Polynorbornenes
Source: Nanomaterials (Basel). 2022 Mar 15;12(6):967. doi: 10.3390/nano12060967 (PMC8951598; doi:10.3390/nano12060967)
Supplement: Supplementary file 1 [file nanomaterials-12-00967-s001.zip › nanomaterials-1573440-supplementary.pdf]

Supplementary Materials

Ultra-high Tg thermoset fibers obtained by electrospinning of functional polynorbornenes

Basile Commarieu, Moubarak CompaoreCompaoré, Raphaël de Boëver, Regis Régis Imbeault, Maxime Leprince, Barbara Martin, Bruno Perard, Weiguang Qiu, and Jerome P. Claverie*

Quebec Center for Functional Materials, Université de Sherbrooke, Department of Chemistry,
2500 Blvd de l'Université, Sherbrooke, QC J1K2R1, Canada*Correspondence: jerome.claverie@usherbrooke.ca; Tel.: +1819-821-8000

Table S1. Solubility of the fibers before and after heat-induced cross-linking

**Fiber**<br><br>**(Expt Table 1)**	**Before curing**	**After cross-linking (200°C)**
**1**	Soluble in DMF, THF	Insoluble in DMF, THF
**2**	Soluble in DMF, THF	Insoluble in DMF, THF
**3**	Soluble in DMF, THF	Insoluble in DMF, THF
**4**	Soluble in DMF, THF	Insoluble in DMF, THF
**5**	Soluble in DMF, THF	Insoluble in DMF, THF
**6**	Soluble in DMF, THF	Insoluble in DMF, THF
**7**	Soluble in water	Insoluble in water and DMF
**10**	Soluble in ethanol, DMF, THF	Insoluble in ethanol, DMF, THF
**12**	Soluble in ethanol DMF, THF	Insoluble in ethanol, DMF, THF

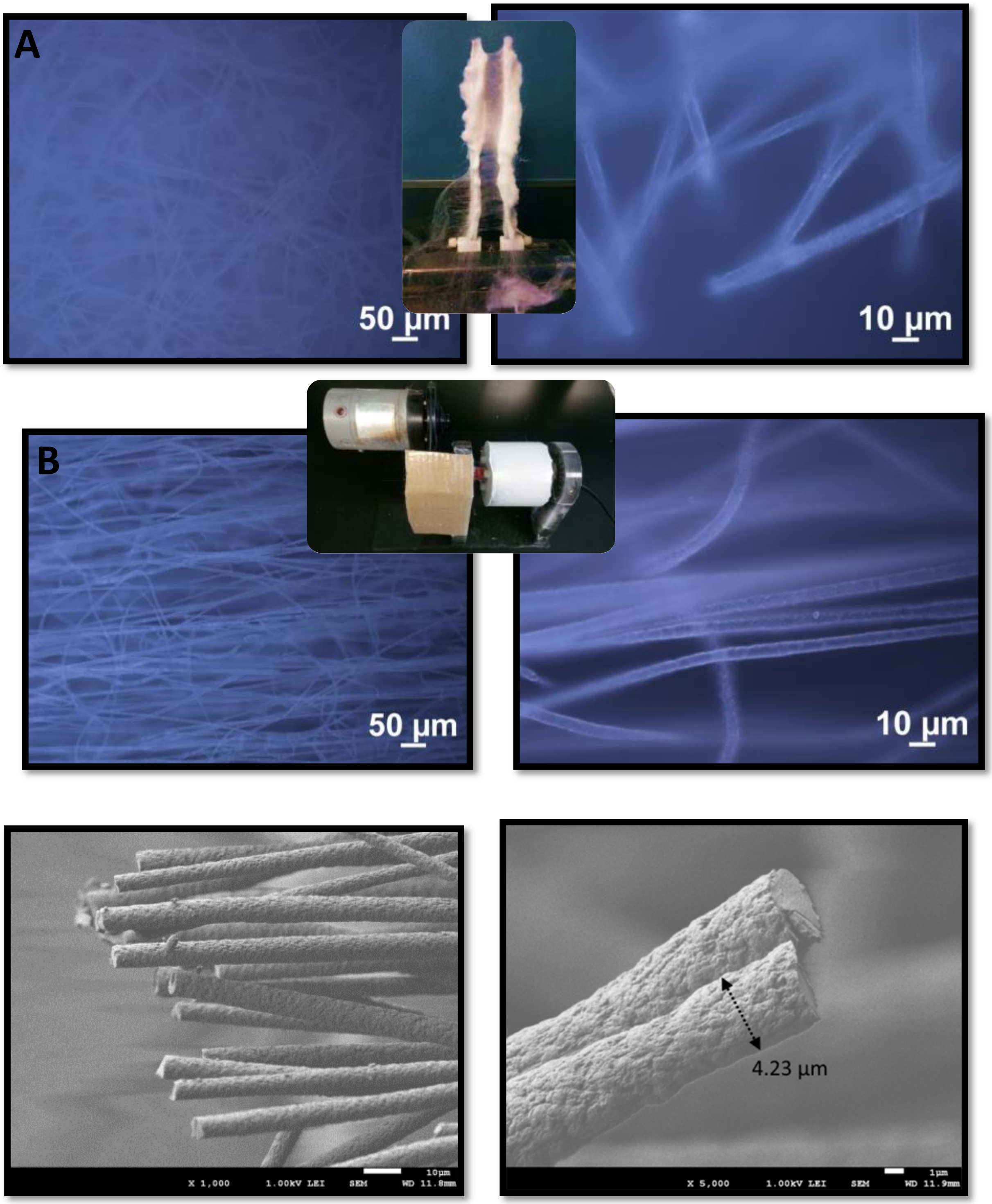

Figure S1. Fibers obtained in experiment 2. A: Picture of the static collector containing fibers and optical microscopy of the corresponding fibers. B: Picture of the rotary collector containing fibers and optical microscopy (top) and SEM (bottom) of the corresponding fibers.

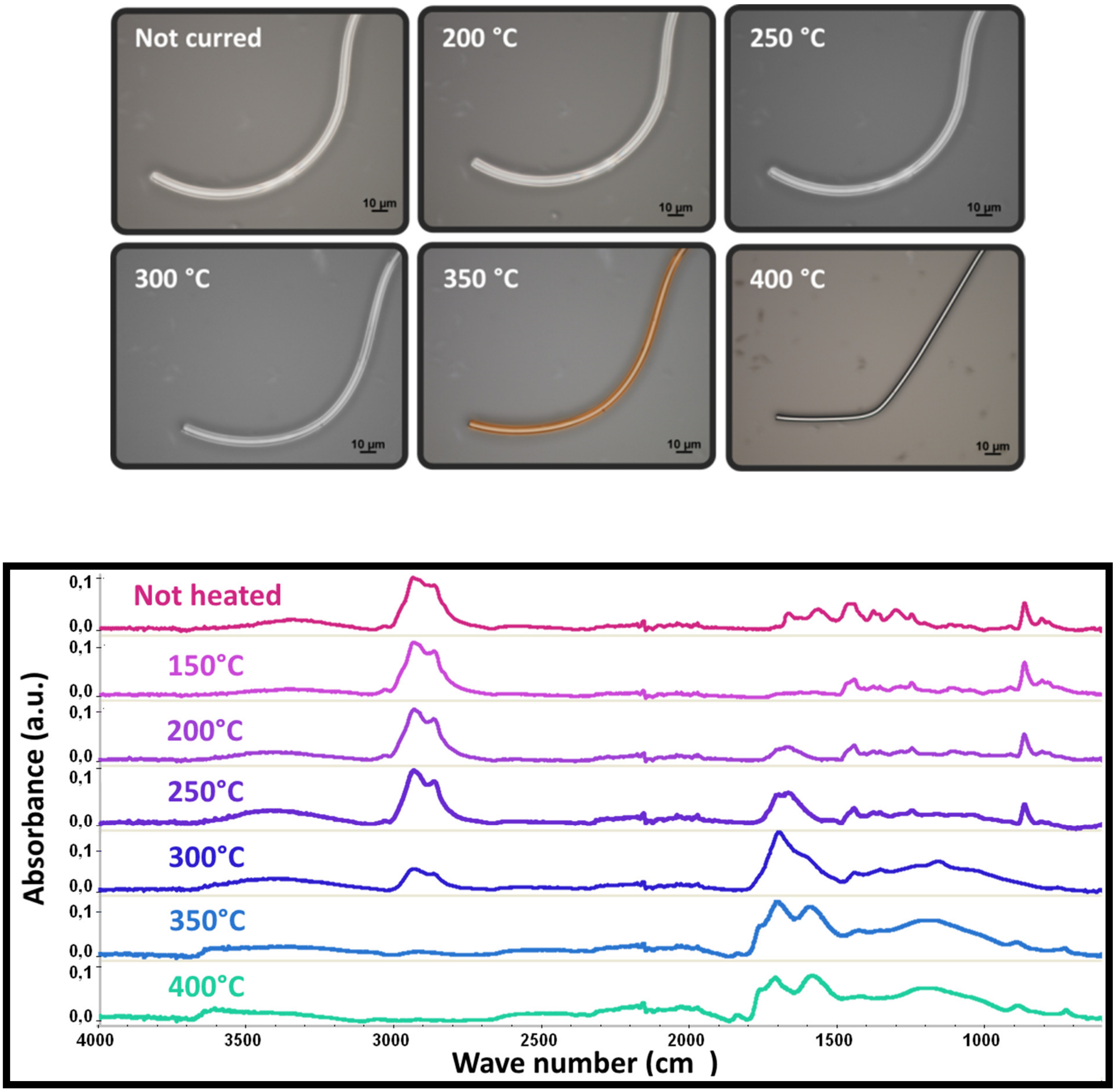

Figure S2. Fibers obtained in experiment 2 before and after heat treatment. Top: optical microscopy. Bottom: FTIR(ATR) analysis. The disappearance of the peak at 847 cm^{-1} corresponds to the ring-opening of the epoxide group.

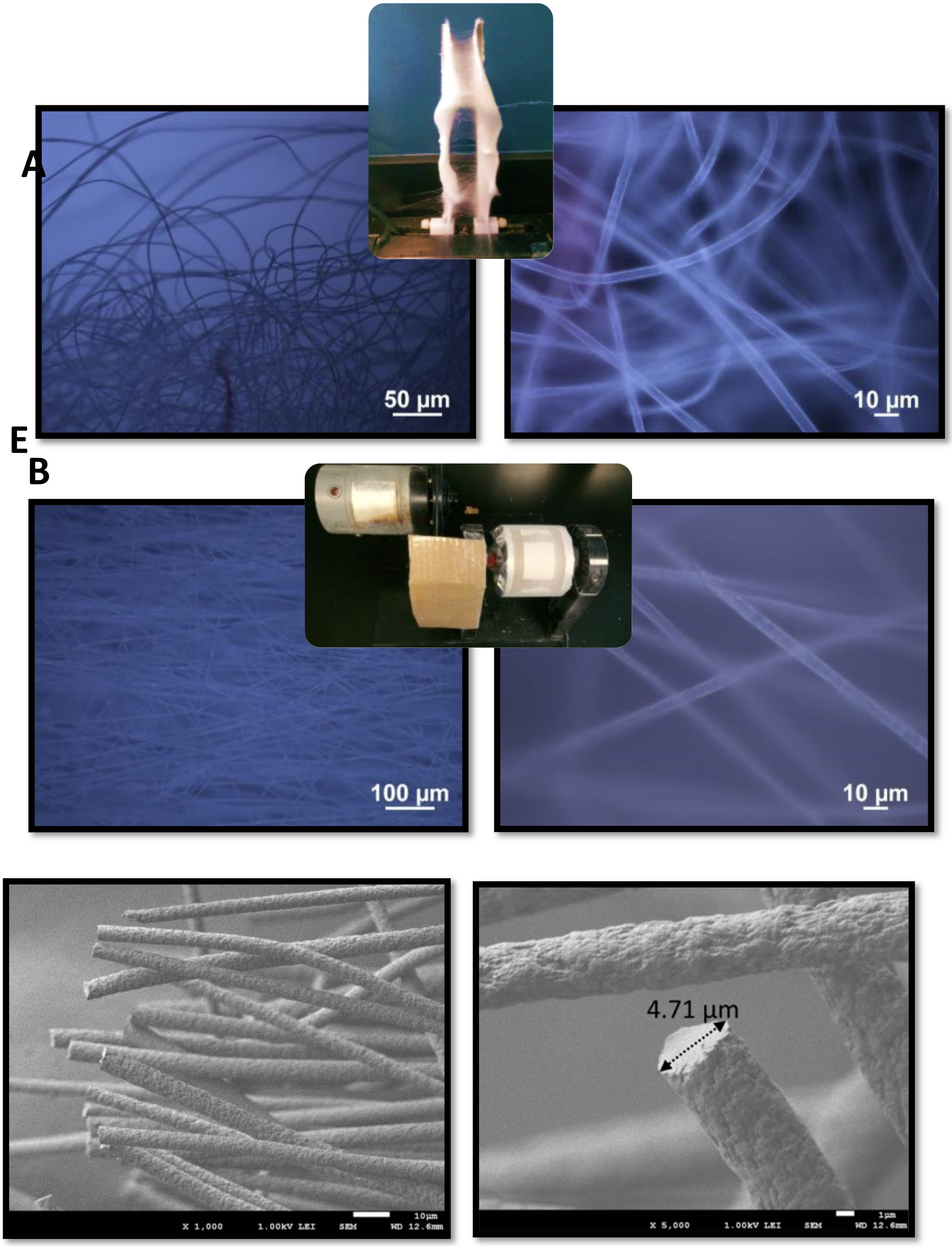

Figure S3. Fibers obtained in experiment 3. A: Picture of the static collector containing fibers and optical microscopy of the corresponding fibers. B: Picture of the rotary collector containing fibers and optical microscopy (top) and SEM (bottom) of the corresponding fibers.

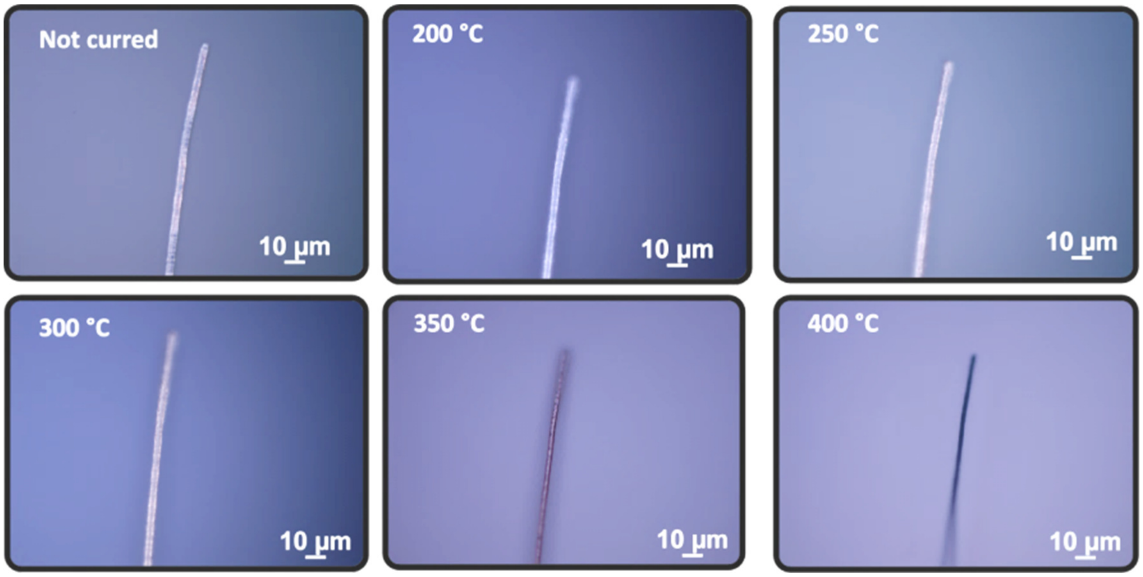

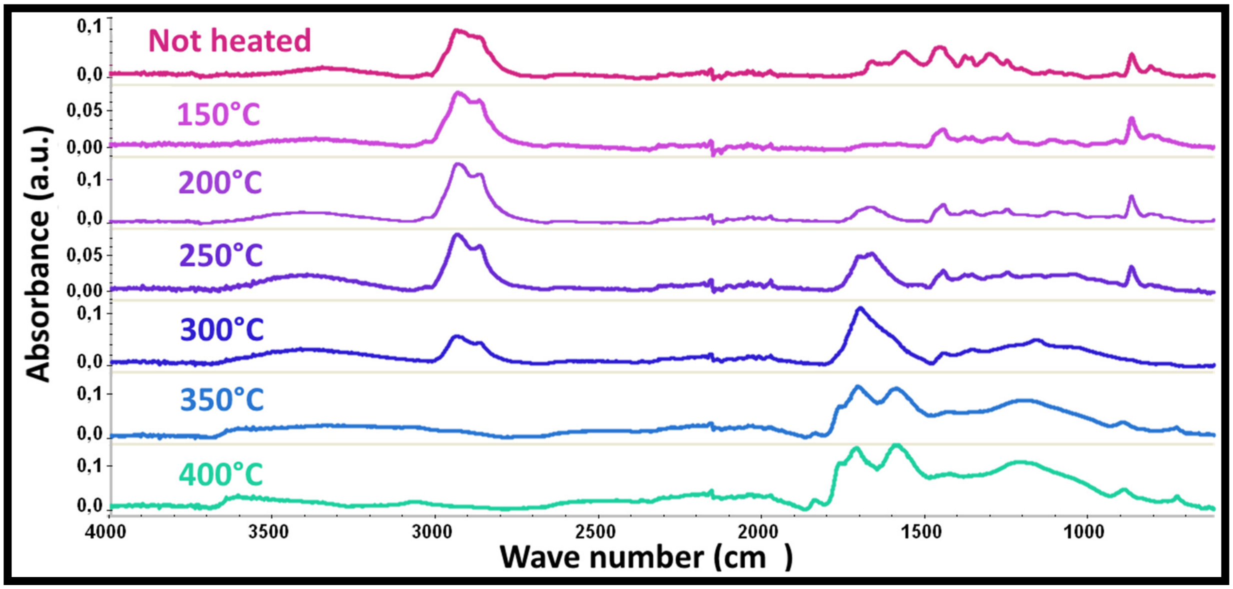

Figure S4. Fibers obtained in experiment 3 before and after heat treatment. Top: optical microscopy. Bottom: FTIR(ATR) analysis. The disappearance of the peak at 847 cm^{-1} corresponds to the ring-opening of the epoxide group.

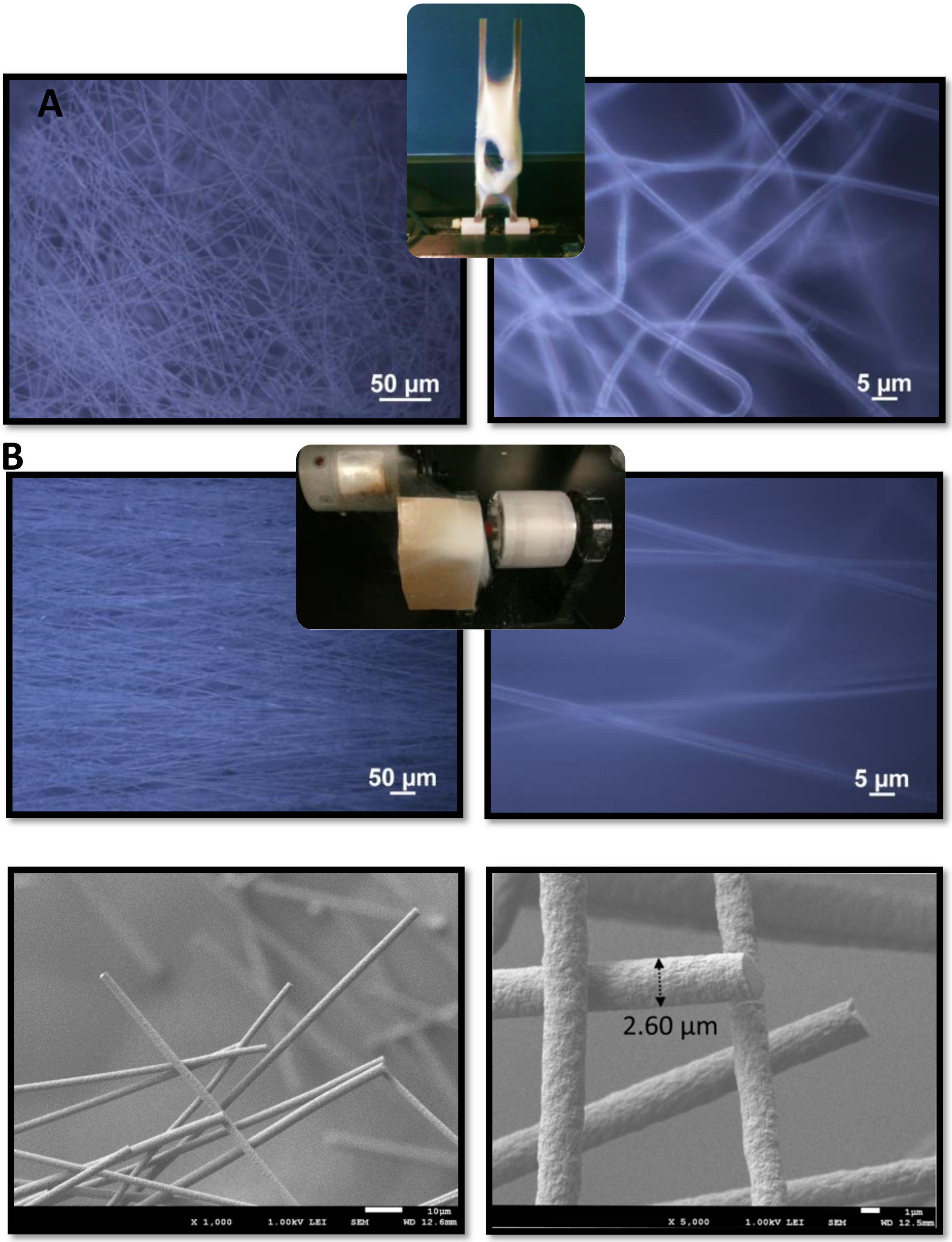

Figure S5. Fibers obtained in experiment 4. A: Picture of the static collector containing fibers and optical microscopy of the corresponding fibers. B: Picture of the rotary collector containing fibers and optical microscopy (top) and SEM (bottom) of the corresponding fibers.

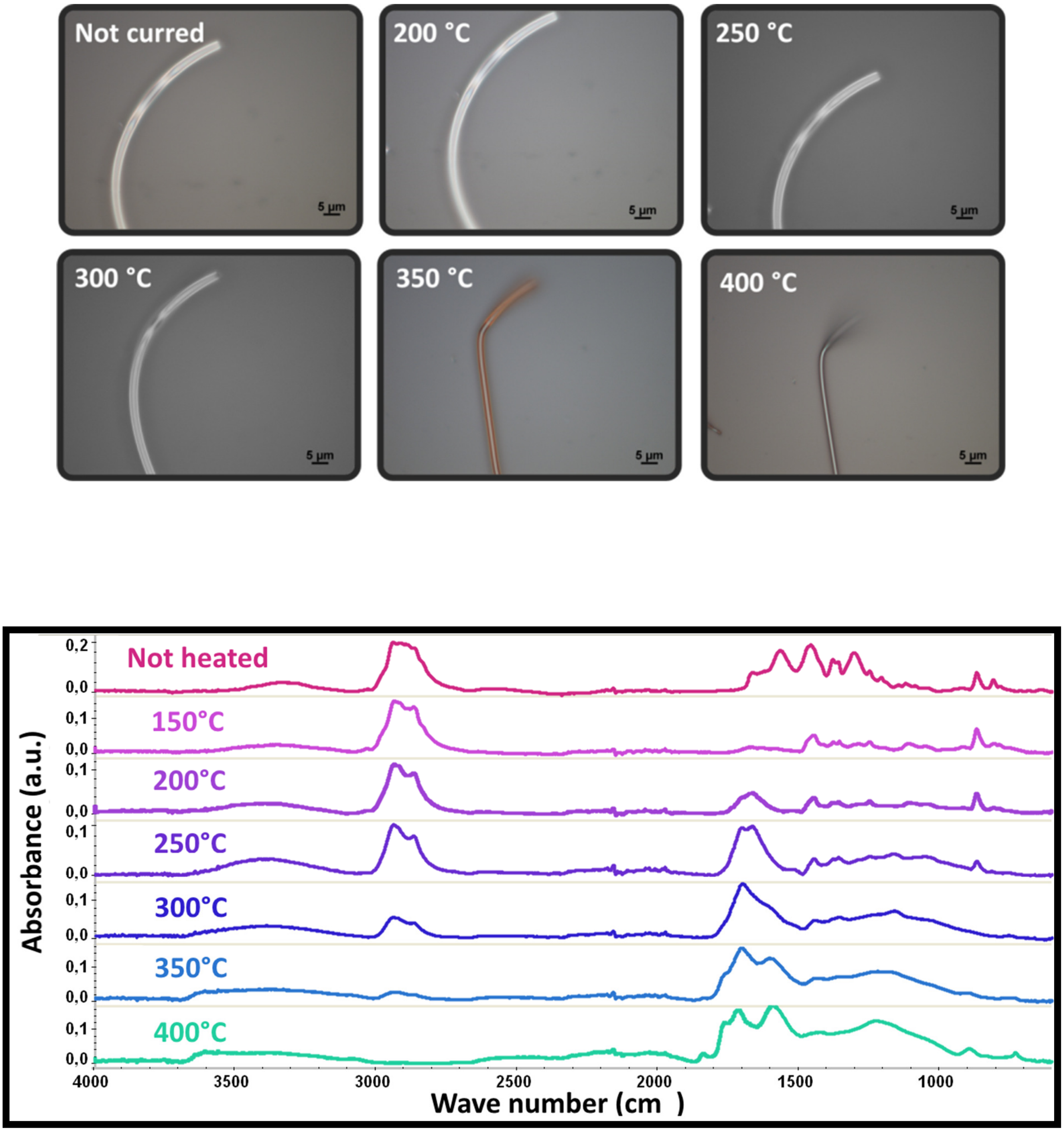

Figure S6. Fibers obtained in experiment 4 before and after heat treatment. Top: optical microscopy. Bottom: FTIR(ATR) analysis. No degradation is observed up to 250 °C. The disappearance of the peak at 847 cm^{-1} corresponds to the ring-opening of the epoxide group.

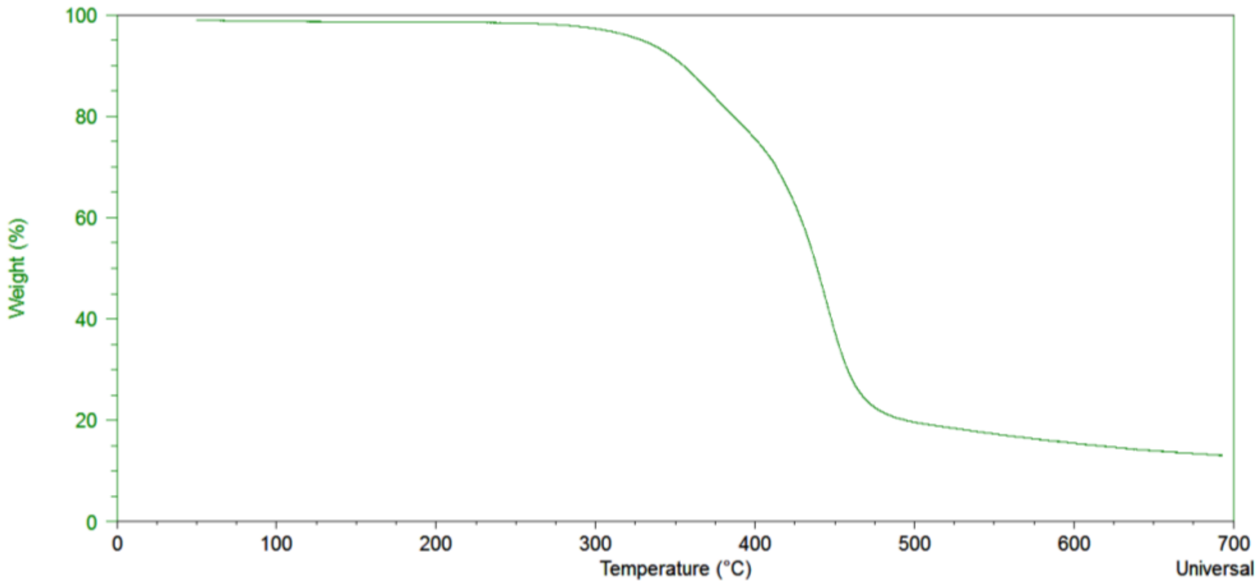

Figure S7. TGA analysis of the fibers prepared in experiment 5.

Expt 7

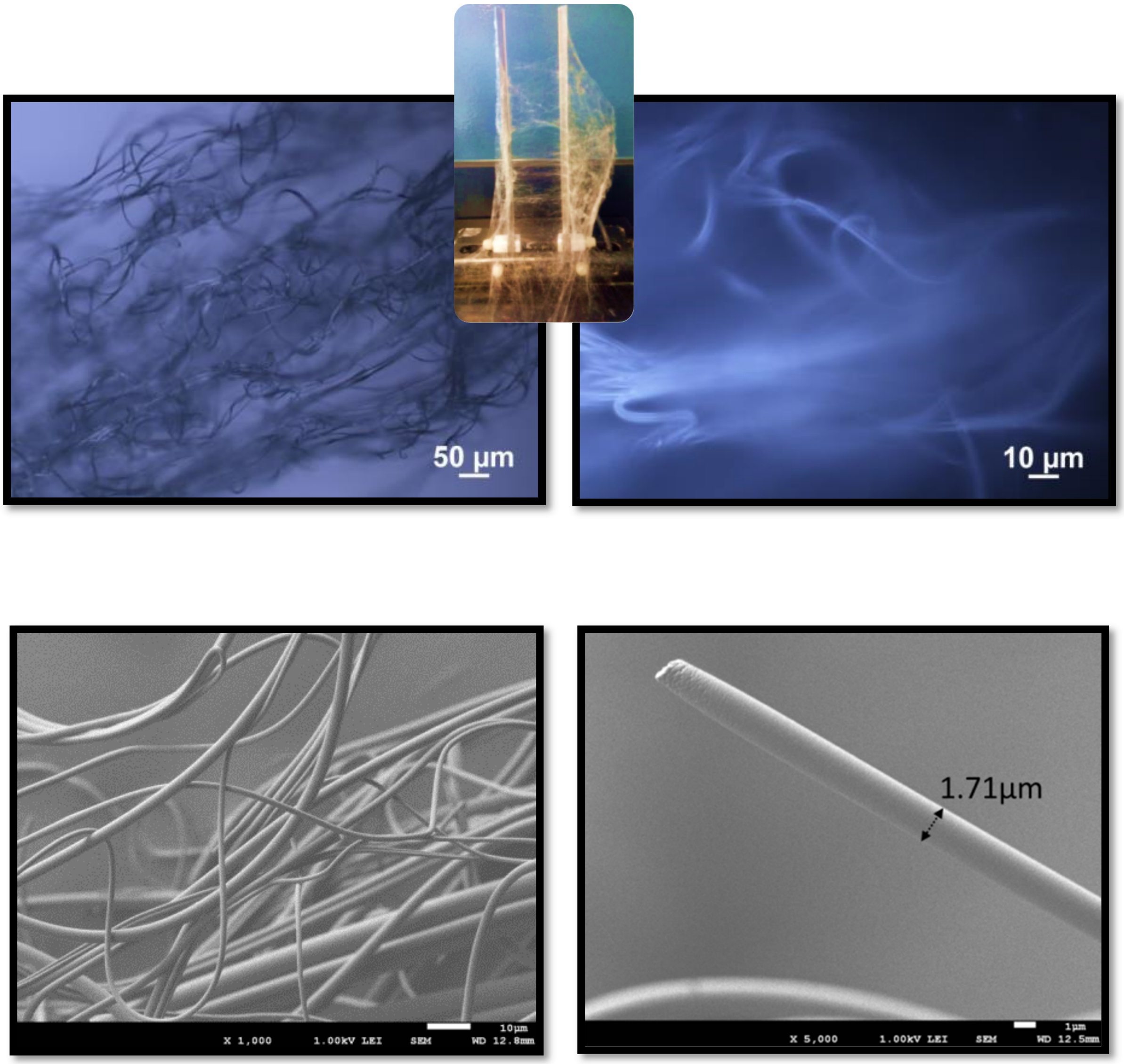

Figure S8. Fibers obtained in experiment 7. Picture of the static collector containing fibers. Optical microscopy (top) and SEM (bottom) of the corresponding fibers.

Expt 7

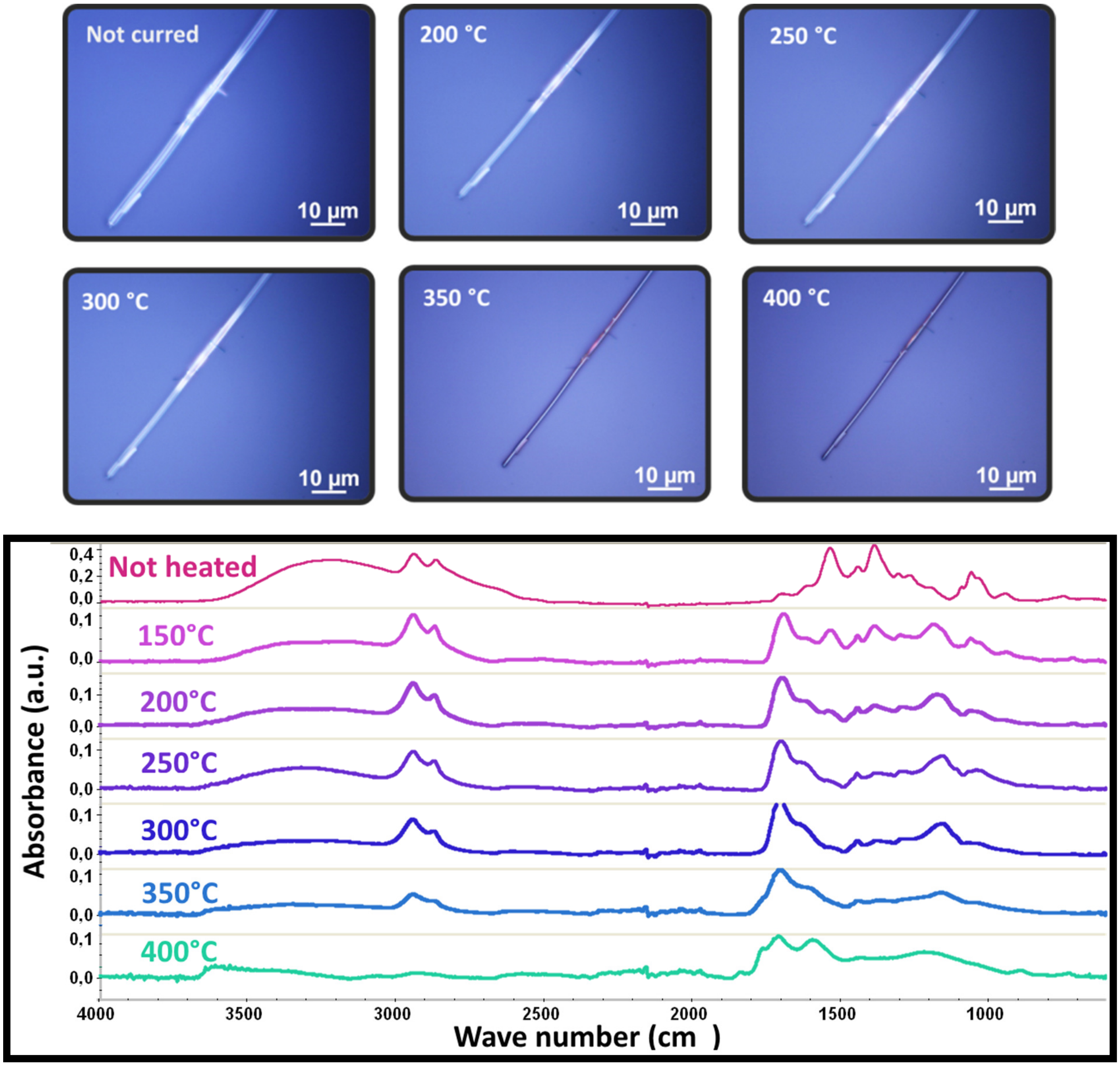

Figure S9. Fibers obtained in experiment 7 before and after heat treatment. Top: optical microscopy. Bottom: FTIR(ATR) analysis.

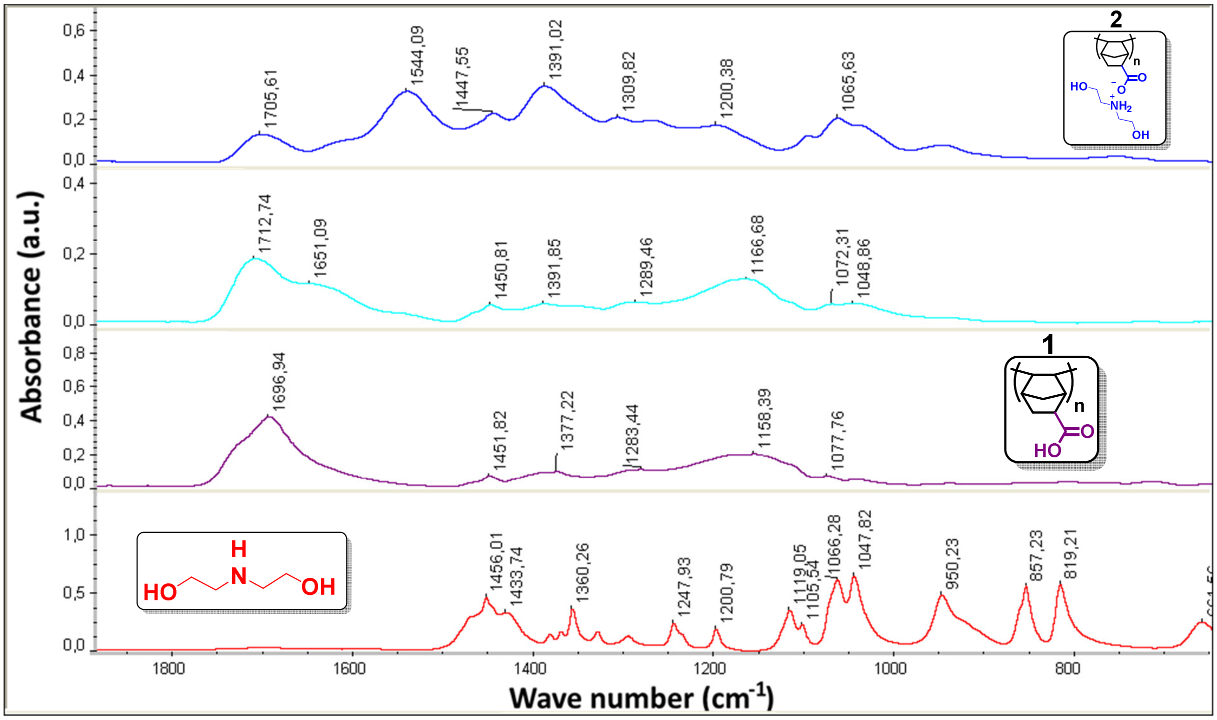

Figure S10. Zoom of the FTIR spectra of (from top to bottom): fibers from experiment 7, fibers from experiment 7 heated at 200 °C for 30 minutes, polymer **2**, diethanol amine.

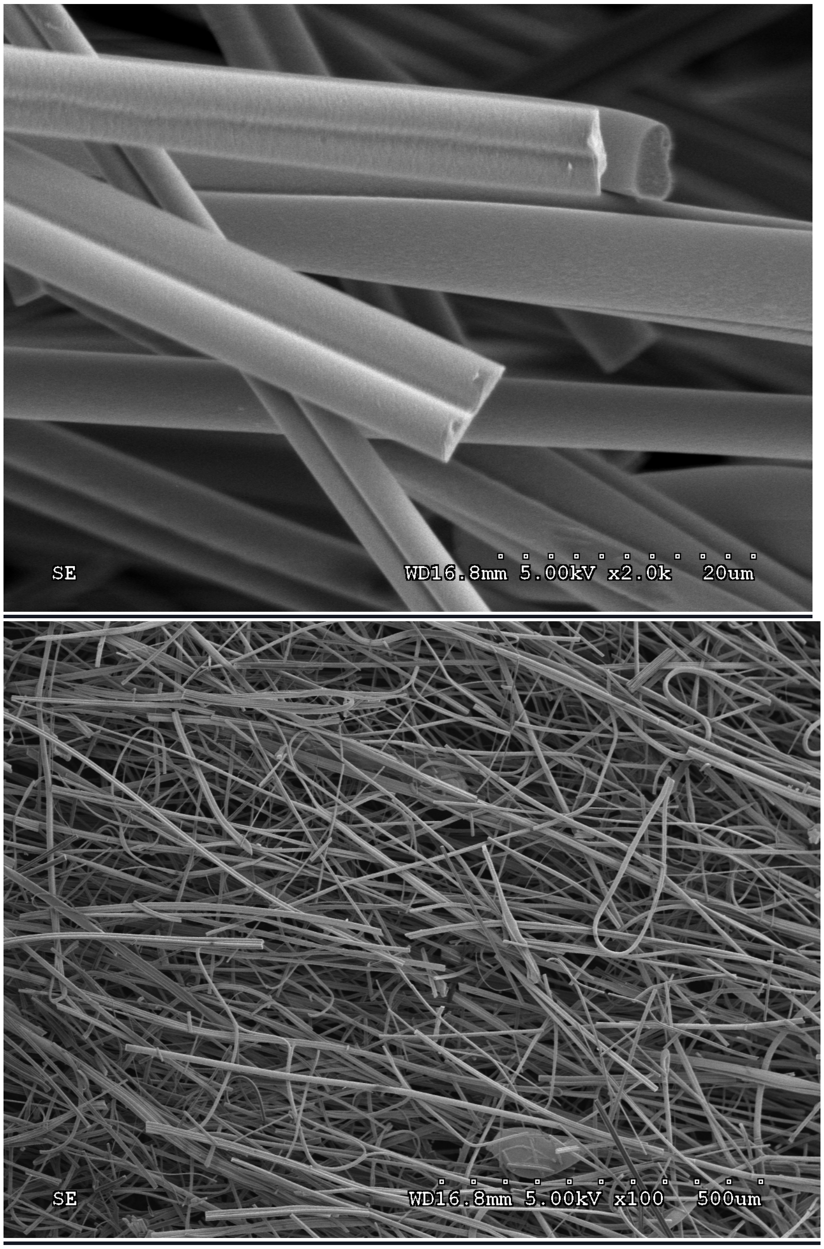

Figure S11. SEM pictures of fibers containing GDs
